# Sex-Specific Associations between Serum IL-16 Levels and Sarcopenia in Older Adults

**DOI:** 10.3390/nu15163529

**Published:** 2023-08-10

**Authors:** Ze Chen, Thea Laurentius, Yvonne Fait, Aline Müller, Eva Mückter, Dandan Hao, Leo Cornelius Bollheimer, Mahtab Nourbakhsh

**Affiliations:** Department of Geriatric Medicine, RWTH Aachen University Hospital, 52074 Aachen, Germany; zchen@ukaachen.de (Z.C.); tlaurentius@ukaachen.de (T.L.); ifait@ukaachen.de (Y.F.); almueller@ukaachen.de (A.M.); emueckter@ukaachen.de (E.M.); dand.hao@outlook.com (D.H.); cbollheimer@ukaachen.de (L.C.B.)

**Keywords:** skeletal muscle, sarcopenia, IL-16, MNA, older adults

## Abstract

Epidemiological studies of older adults have suggested a differential sex-specific prevalence of sarcopenia, which is a condition characterized by a progressive loss of skeletal muscle mass and function. Recently, we collected serum samples from 80 fully evaluated older adults and identified CXCL12α as a sex-independent serum marker of sarcopenia. Here, we used this serum collection to find potential sex-specific serum markers via the simultaneous quantification of 34 inflammatory cytokines/chemokines. The appendicular skeletal muscle index (ASMI) was used as a decisive criterion for diagnosing sarcopenia. A Pearson correlation analysis revealed a negative correlation between ASMI and serum IL-16 in females only (*p* = 0.021). Moreover, women with sarcopenia exhibited significantly higher IL-16 (*p* = 0.025) serum levels than women in a control group. In contrast, males with sarcopenia had lower IL-16 (*p* = 0.013) levels than males in a control group. The further use of Fisher’s exact test identified obesity (*p* = 0.027) and high serum levels of IL-16 (*p* = 0.029) as significant risk factors for sarcopenia in females. In male older adults, however, malnutrition (*p* = 0.028) and low serum levels of IL-16 (*p* = 0.031) were the most significant risk factors for sarcopenia. The differential sex-specific associations of IL-16 in older adults may contribute to the development of more precise regression models for future research and elucidate the role of IL-16 in the progression of sarcopenic obesity.

## 1. Introduction

Reductions in skeletal muscle mass and strength are a natural part of the aging process. When this process exceeds the normal range, as demonstrated by a combination of factors, it meets the diagnostic criteria for sarcopenia [1,2]. Thus, the etiology of sarcopenia can encompass several factors, including obesity, malnutrition, physical inactivity, and chronic diseases, such as diabetes, liver disease, and kidney disease [3,4,5]. The clinical manifestations of sarcopenia include reductions in muscle mass and muscle quality, diminished physical strength, impaired mobility, decreased metabolic rate, and increased susceptibility to osteoporosis. The diagnosis of sarcopenia in older adults typically relies on a clinical assessment and measurements of muscle mass, strength, and physical function. The widely accepted assessment tools include the measurement of muscle mass using a bioelectrical impedance analysis (BIA) or dual X-ray absorptiometry (DXA) and assessments of grip strength and functional mobility [1]. However, each method has its own limitations in terms of accuracy or complexity. To improve the diagnostic accuracy and reproducibility of sarcopenia in older adults, researchers have been actively searching for reliable biomarkers. These biomarkers can include specific molecules in the blood, metabolic byproducts, or changes in the expression of muscle-related genes [6,7].

Cytokines and chemokines are a class of signaling molecules that play crucial roles in regulating biological processes, including the immune response, inflammation, and tissue regeneration. The abnormal activation of the immune system and inflammation were suggested to enhance the breakdown of muscle proteins and thereby contribute to the pathogenesis of sarcopenia [8]. Recent research indicates that alterations in hormone levels and the enhanced expression of cytokines and chemokines may contribute to the development of sarcopenia [9,10,11]. These findings strongly suggest that the development of chronic inflammation may be closely associated with the progression of sarcopenia in older adults. Thus, the modulation of cytokine levels holds promise as a strategy for preventing and treating sarcopenia, and certain medications, such as anti-inflammatory drugs and immunosuppressants, may help to improve the prognosis for patients with sarcopenia.

The chemokine IL-16 was initially implicated in human T-cell activation and chronic inflammatory diseases, such as rheumatoid arthritis [12]. In patients with sepsis, IL-16 was implicated in promoting inflammation in cardiomyocytes via a synergistic interaction with TNF-α, IL-1β, and IL-6 [13]. High serum IL-16 levels were associated with the development and progression of malignancy and the poor survival of patients suffering from gastric cancer and sarcopenia [9]. Gene polymorphisms that elevate IL-16 expression were associated with increasing serum IL-16 levels and a significantly higher risk of developing gastric cancer in men and women equally [14]. IL-16 levels may also be regulated by hormones, as recent research reported that the expression of IL-16 can change in women due to ovarian aging [15].

Nutrition plays a dual role in the development of sarcopenia. On one hand, malnutrition is a significant contributing factor to age-related sarcopenia [1]. An inadequate intake of protein and energy due to prolonged malnutrition can lead to muscle wasting and a decline in muscle mass [16,17,18]. On the other hand, excessive nutrition can contribute to sarcopenia through obesity. Obesity can lead to declines in muscle mass and strength, thus promoting sarcopenia. In addition, the accumulation of fat in muscle tissue, known as “fat infiltration,” occurs in individuals with obesity. This condition, known as sarcopenic obesity (SO), is associated with metabolic disorders, chronic inflammation, and impaired muscle strength and function [19,20,21].

Recently, we studied an inclusive set of clinical data and serum samples from 80 older individuals who were of Northern European descent and underwent a series of geriatric assessments. In the first attempt, we found a negative correlation between skeletal muscle mass in older adults and the serum level of CXCL12α, a chemoattractant for muscle stem cells. Here, we quantified the levels of an additional 34 cytokines/chemokines in our collected serum samples to identify potential sex-specific markers for sarcopenia. STROBE reporting guidelines (https://www.strobe-statement.org (accessed on 8 August 2023) were followed, and anonymized study data were made available at the following website: https://www.ukaachen.de/sarkopenie (accessed on 8 August 2023).

## 2. Materials and Methods

### 2.1. Study Participants

Eighty consecutive inpatients were included in this cross-sectional study between February and December 2019. All study participants were of Northern European descent and met the following criteria: over 65 years of age, willing to participate voluntarily, and capable of following simple instructions and walking 10 m with or without a walking aid. Patients with the following criteria were excluded: having an implantable cardioverter defibrillator or pacemaker, severe visual impairment, serious statin-induced myopathy, cardiovascular disorders, or other complications that would hinder their participation in the study.

### 2.2. Collection and Handling of Serum Samples

Fasting blood samples were obtained via venipuncture and collected in collection tubes (BD Vacutainer^®^; Becton, Dickinson and Co., Franklin Lakes, NJ, USA). One aliquot of the blood samples was sent to a certified laboratory (MVZ Labor, Bochum, Germany) for measurements immediately after sampling. Another aliquot of the blood samples was incubated at room temperature for 20 min and centrifuged to obtain the serum fraction. Multiple aliquots of the serum samples were stored at −80 °C for further analyses.

### 2.3. Measurement of Candidate Biomarkers

The serum levels of the human inflammatory markers TNFSF13, TNFSF13B, BDNF, GDF2, EGF, FABP-3, CNPY2, ILCF, HGF, IL-2R, IL-13, IL-16, IL-20, LIF, CCL7, CCL8, CCL24, CXCL9, ALOX15, MDC, MIF, MMP-1, MMP-3, MMP-9, MMP-13, NGF β, PDGF-BB, PlGF-1, SCF, TNF-RII, ICAM-1, VCAM-1, VEGFA, and VEGF were assessed using a ProcartaPlex Human Cytokines/Chemokines Panel (Thermo Fisher Scientific, Waltham, MA, USA). All samples were simultaneously prepared and analyzed according to the instructions of the manufacturer. Readings that were lower than the assay detection limit or three-fold higher than the standard deviation of the mean (outliers) were excluded. The mean values of four parallel measurements were calculated as the final concentration of each marker and are presented in the Supplementary Materials, Table S1.

### 2.4. Analysis of the Appendicular Skeletal Muscle Mass

The appendicular skeletal muscle mass (ASMM) was estimated using a Bioelectrical Impedance Analysis (BIA) device (BIACORPUS RX 4004 M; MEDI CAL Healthcare GmbH, Karlsruhe, Germany). An integrated software based on the Sergi equation was utilized to calculate the appendicular skeletal muscle index (ASMI), as described in the manufacturer’s instructions. The participants were ensured to be well hydrated and instructed to not eat 3–4 h before the measurements were taken. The participants were instructed to rest in a standard hospital bed (in a supine position with a maximum head inclination of 30°) for 5 min. A standard disinfecting towel was used to moisturize the participant’s hands and heels. Then, two electrodes were placed on each extremity. Four electrodes were placed between the medial and lateral malleoli and on the upper border of an imaginary line between the radius and ulna head. Another four distal electrodes were placed at a distance of 5 cm from the proximal electrodes. The measurements were performed bilaterally when possible. During the measurements, the room temperature and humidity were maintained at 18–22 °C and 50–60%, respectively.

### 2.5. Grip Strength Measurement

Hand grip strength was measured using a SAEHAN DHD-1 digital hand dynamometer (SAEHAN Corporation, Masan, Republic of Korea). Each participant was instructed to sit upright on a chair with their elbow entirely resting on the armrest (90°) and to stably place their feet on the ground. Then, the dynamometer was placed and adjusted to the participant’s dominant hand. After the adjustments, the participant was instructed to press the dynamometer as strongly as possible (maximum strength). Each assessment was repeated 3 times to obtain a mean value (kg).

### 2.6. Physical Assessment and Evaluation

The physical, cognitive, and physiological health statuses of the participants were assessed by trained personnel (physicians, nurses, physiotherapists, occupational therapists, or dietitians) using standardized assessment tools within 48 h after admission. We referred to the new sarcopenia diagnostic criteria released by the EWGSOP in 2019 and, based on the actual situation of the study sample and the results of the pre-analysis data, raised the sarcopenia standard for this study. The diagnostic standard for sarcopenia in this study was described previously [22]. Briefly, females with an ASMI < 6.8 kg/m^2^ and males with an ASMI < 7.3 kg/m^2^ were classified as the patient group. The control group included females with an ASMI ≥ 6.8 kg/m^2^ and males with an ASMI ≥ 7.3 kg/m^2^.

### 2.7. Statistical Analysis

The statistical analyses and the presentation of data were carried out using SPSS 21.0 software (IBM Corp., Armonk, NY, USA). Descriptive statistic tools were utilized to characterize the study groups and to assess the normal distribution of the ASMI scores. The means of continuous variables were expressed as means ± standard deviation (SDs). Categorical variables were shown in counts or percentages via 95% confidence intervals (CIs). The associations between the ASMI and other variables were determined using a Pearson correlation analysis. An independent samples *t*-test was used to test the significance (*p*) of the differences between the normally distributed variables in the groups. The Wilcoxon rank-sum test was used to test the differences between the variables that did not follow a normal distribution in the study groups. The differences between categorical variables were assessed using Fisher’s exact test. The odds ratio (OR) was used to measure the strength of the association between the exposure factor and the disease factor. In the entire study, we applied a significance level of 0.05, and a *p*-value less than 0.05 was considered statistically significant. The 3D data models were produced using OriginLab 2019b.

## 3. Results

### 3.1. ASMI and Serum Levels of IL-16 Correlate Exclusively in Older Females

The general characteristics of the study participants, 80 older adults between 69 and 93 years of age, were described previously [22]. Table 1 summarizes the participant characteristics that are relevant to the current study. The mean values of the ASMI, weight, and grip strength in males were significantly higher than those in females. In agreement with previous epidemiological observations, the mean body fat ratio (BFR) was significantly higher in older females. However, we found no significant sex-specific differences in age, MNA score, or IL-16 serum level (Table 1).

**Figure 1:**
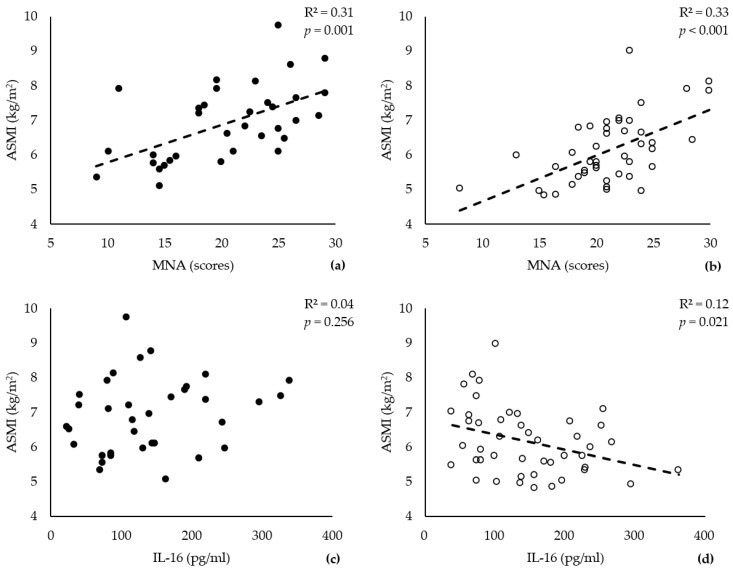
ASMI and serum levels of IL-16 correlate exclusively in older females. Dot plots show the correlations between the ASMI and MNA scores (**a**,**b**) or IL-16 serum levels (**c**,**d**) in males (*n* = 35, black circles) and females (*n* = 45, white circles). Pearson’s correlation coefficient (R^2^) and the significance (*p*) are provided at the top of each graph. Variables and their units are indicated on the corresponding axes.

A Pearson correlation analysis was used to examine the strength of the linear relationships between the ASMI and the MNA score or the serum IL-16 level. The data showed a significant positive correlation between the ASMI and the MNA score in both males and females (Figure 1a,b, males: r = 0.560, *p* = 0.001; females: r = 0.571, *p* < 0.001). However, only the female group showed a significant negative correlation between the ASMI and the IL-16 level (Figure 1d; r = −0.342, *p* = 0.021). We observed a trivial positive correlation between the IL-16 level and the ASMI in males, although it was not significant (Figure 1c, r = 0.179, *p* = 0.256).

### 3.2. BFR and IL-16 Are Influencing Factors in Older Female Patients with Sarcopenia

Using statistical models, we verified the ASMI cutoffs for the sarcopenia patient (male < 7.3 kg/m^2^ and female < 6.8 kg/m^2^) and control (Male ≥ 7.3 kg/m^2^ and female ≥ 6.8 kg/m^2^) groups in our previous study [22]. The characteristics of the sarcopenia patient and control groups divided by sex are summarized in Table 2. By comparison, the patient and control groups showed no significant differences in the participants’ sex or age. However, there were significant differences in weight, ASMM, and IL-16 level between the patient and control groups. In female participants specifically, we found substantial differences in the mini nutritional assessment (MNA) scores or body fat ratio (BFR) and no significant difference in fat mass (FM) between the case and control groups (Table 2).

### 3.3. IL-16 Serum Levels and BFRs Are Significantly Higher in Older Females

**Figure 2:**
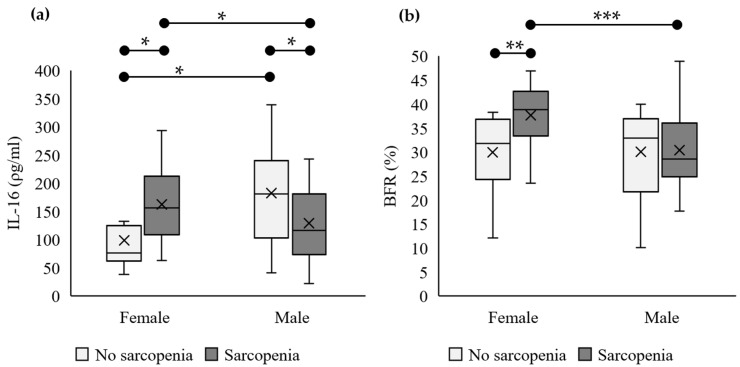
IL-16 serum levels and BFRs are significantly higher in older females. Box plots show the mean (×) and median (75th or 25th percentile, horizontal line) IL-16 serum levels (**a**) and BFRs (**b**) in males and females without sarcopenia (light gray) or with sarcopenia (dark gray). * *p* < 0.05, ** *p* < 0.01 or *** *p* < 0.001.

The box plots in Figure 2 provide further descriptions of the results in Table 2. The IL-16 levels in the female patient group were significantly higher than those in the control group, while the opposite was true for males. Within the patient group, females had significantly higher IL-16 levels than males, while the opposite was true for the control group (Figure 2a). The box plot for the BFR shows indistinguishable results, with significantly higher BFRs in the female patient group than in the control group but no significant difference in means between the male patient and control groups (Figure 2b).

### 3.4. High IL-16 Serum Levels Serve Exclusively as a Risk Factor for Sarcopenia in Older Females

We used a multivariate analysis to estimate the sex-specific risks for developing sarcopenia depending on multiple indicators. Therefore, Fisher’s exact test was performed to calculate the intergroup differences between the patients and the controls. The results are summarized in Table 3. The calculated odds ratios (ORs) reflect the strength of the association between the designated characteristics and the patient group. Three indicators were included in this model: MNA, BFR, and IL-16 serum level. Males with low MNA scores had a significantly higher risk for sarcopenia (OR: 9.75, 95% CI: 1.07, 88.87, *p* = 0.028) than females. In contrast, obese females exhibited a significant risk for sarcopenia (OR: 5.83, 95% CI: 1.25, 27.17, *p* = 0.027) compared to lean females. Most importantly, the data indicate discordant risks for sarcopenia in males and females with IL-16 serum levels higher than 150 pg/mL (OR: 9.53, 95% CI: 1.09, 83.44, *p* = 0.029). In females, the OR for sarcopenia was more than ninefold higher with IL-16 serum levels over 150 pg/mL (OR: 9.52, 95% CI: 1.09, 83.44, *p* = 0.029). In males, however, the OR for sarcopenia was significantly higher for IL-6 levels lower than 150 pg/mL (OR: 0.18, 95% CI: 0.04, 0.81, *p* = 0.031). Taken together, IL-16 serum levels may serve as an indicative marker for sarcopenia in male and female adults differentially. Furthermore, the data highlight a possible involvement of IL-16 in the sex-specific pathogenesis of sarcopenia in older adults.

### 3.5. BFR and MNA Correlate in Women with Sarcopenia with High IL-16 Serum Levels

**Figure 3:**
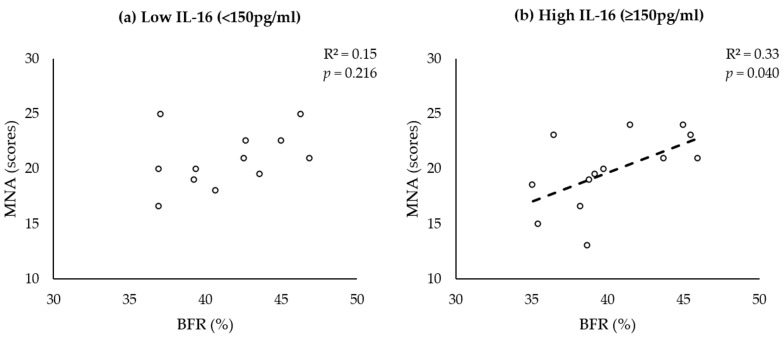
BFR and MNA correlate with high IL-16 serum levels in women with sarcopenia. Dot plots show the correlations between the BFR (*x*-axis) and MNA score (*y*-axis) in women with sarcopenia (white circles) with low ((**a**), *n* = 12) or high IL-16 serum levels ((**b**), *n* = 13). Pearson’s correlation coefficient (R^2^) and the significance (*p*) are provided at the top of each plot.

The multivariate analysis of female participants revealed that high IL-16 levels, low MNA scores, and obesity contribute to a higher risk of sarcopenia (Table 3). This led to the assumption that MNA and obesity may correlate in females with high IL-16 serum levels. We performed Pearson’s correlation analysis and included all women with sarcopenia. The results in Figure 3 demonstrate a significant correlation between the BFR and MNA score exclusively in women with sarcopenia with high IL-16 levels.

### 3.6. High IL-16 Serum Levels Are the Primary Factor Influencing the Probability of Sarcopenia in Older Females

**Figure 4:**
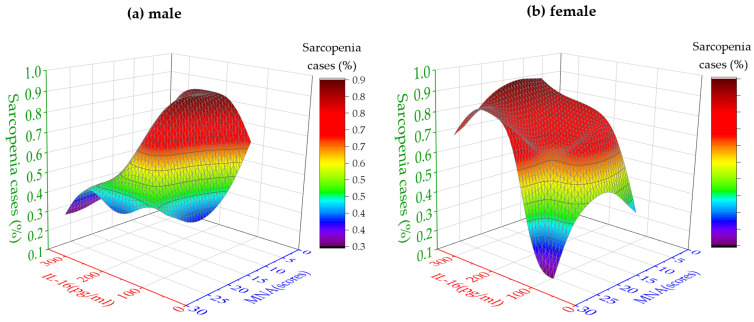
High IL-16 serum levels are the primary factor influencing the probability of sarcopenia in older females. The 3D diagrams show the percentage of sarcopenia cases (*y*-axis, color scale) based on MNA scores (*z*-axis, scores) and IL-16 serum levels (*x*-axis, pg/mL) in males (**a**) and females (**b**).

Because the sex-specific risk factors for sarcopenia were the focus of this study, we systematically calculated the share of sarcopenia patients (percentage) via increasing variables, serum IL-16 levels, and MNA scores. The data are summarized in Figure 4 by two 3D contour histograms for male (Figure 4a) and female (Figure 4b) participants. The histograms reflect the actual variations in all samples more clearly. Taken together, over 80% of men with sarcopenia had lower MNA scores (Figure 4a). This corresponds to the data in Table 3, indicating that the risk for sarcopenia was particularly high when MNA scores were less than 17 (OR: 9.75, 95% CI: 1.07, 88.87, *p* = 0.028). In other words, good nutritional status may be a protective factor for sarcopenia in older men. In sharp contrast to the male participants, almost 90% of the females with sarcopenia had elevated IL-16 serum levels. Consistent with the data in Table 3, IL-16 likely has a greater impact on the probability of sarcopenia in older females than on their nutritional status. Moreover, a high IL-16 serum level ≥150 pg/mL was the most significant risk factor for sarcopenia in older females (OR: 9.53, 95% CI: 1.09, 83.44, *p* = 0.029). Thus, improving the nutritional status in females may have a minor effect on the risk for sarcopenia.

## 4. Discussion

Clinical research on older adults allows us to gain a comprehensive understanding of the interplay between sarcopenia and inflammatory factors, elucidate the underlying mechanisms driving sarcopenia, and provide scientific evidence for its prevention and treatment. Potential correlations between sarcopenia, inflammation, and multiple cytokines or chemokines have been extensively studied in the fields of geriatrics and muscle biology [23]. Our study identified IL-16 and nutritional status as significant contributing factors to decreases in muscle mass and strength in older adults. Moreover, the impact of IL-16 on sarcopenia underlies sex differences; therefore, the serum IL-16 level may serve as a potential biomarker for sarcopenia in older women. However, nutritional status was more relevant to the development of sarcopenia in older men. These outcomes can help delay muscle aging and improve health status and physical function in older adults. Our data contribute to the existing knowledge in the field of sarcopenia and provide valuable insights for future research and clinical applications.

The findings of this study indicate a significant positive relationship between an improved nutritional status (MNA score) and enhanced muscle mass in older adults irrespective of sex. Indeed, the MNA scores were strongly correlated with the ASMI in males and females (males: r = 0.560, *p* = 0.001; females: r = 0.571, *p* < 0.001). The impact of nutrition on aging muscle mass is indisputable [24]. Adequate dietary nutrition in the older population ensures sufficient energy and essential nutrients, thereby promoting muscle protein synthesis and maintenance [25]. However, in our study, the in-depth analysis of Fisher’s exact test (Table 3) and 3D probability model (Figure 4) revealed a more prominent effect of nutritional status on muscle mass in males than in females. Malnutrition emerged as a significant risk factor for sarcopenia in males (OR: 9.75, 95% CI: 1.07, 88.87, *p* = 0.028), while no significant association was observed in females.

The impact of IL-16 on muscle condition in males and females yielded divergent outcomes. We found a significant negative correlation between the IL-16 level and ASMI in females (Figure 1d, r = −0.342, *p* = 0.021) and identified elevated serum IL-16 as a risk factor for sarcopenia in females (OR: 9.53, 95% CI: 1.09, 83.44, *p* = 0.029). Thus, older females with better muscle mass generally exhibit lower levels of IL-16. Conversely, in males, the Pearson correlation analysis did not reveal a significant association between the IL-16 level and ASMI, suggesting the absence of a pronounced linear relationship. However, both Fisher’s exact test and the 3D probability model identified a high IL-16 level as a protective factor against sarcopenia in males, yielding statistically significant results (OR: 0.18, 95% CI: 0.04, 0.81, *p* = 0.031). The discrepancy between the Pearson correlation analysis and the findings of Fisher’s exact test and the 3D probability model in males may be attributed to the relatively small sample size of males (*n* = 35), the potential presence of a nonlinear correlation between the IL-16 level and ASMI, and the concurrent influence of other cytokines on the results.

Further research on the effects of the BFR in both the patient and control groups revealed that according to the World Health Organization’s obesity standards (BFR > 25% for males and BFR > 35% for females), the males in both groups were classified as obese, with no statistically significant differences in BFRs. However, the BFRs of the females in the patient group were significantly higher than those of the females in the control group (*p* = 0.002), and they surpassed the obesity threshold. Figure 2 demonstrates a consistent pattern in which the females’ BFRs corresponded with the levels of IL-16, with the patient group exhibiting significantly higher values than those in the control group. Fisher’s exact test indicated that female obesity was a risk factor for sarcopenia (OR: 5.83, 95% CI: 1.25, 27.17, *p* = 0.027), whereas there was no direct association between male obesity and sarcopenia. Based on Table 1, there was no significant difference in nutritional status between the males and females (*p* = 0.782). However, among the females, the patient group displayed a significantly higher body fat percentage than the control group, reaching the obesity threshold, despite having a relatively lower body weight.

By analyzing the data presented in Table 2, we calculated the differences between the female control and patient groups for the ASMM (4.72 kg), FM (−1.88 kg), and weight (9.78 kg). For males, the corresponding differences were 5.57 kg, 4.64 kg, and 17.65 kg, respectively. Thus, both the female and male patient groups showed decreases in ASMM and weight values compared with those of their respective control groups. In addition, only the male patient group showed a decrease in FM, while the female patient group displayed an increase in FM. Consequently, the magnitude of the ASMM decrease in females is 2.51 times the magnitude of the FM increase, whereas in males, the ASMM decrease is 1.2 times the magnitude of the FM decrease. Considering the overall body weight loss within the patient group, the rate of muscle mass loss was significantly higher in females (48%) than in males (32%), as determined by the ASMM loss/weight loss ratio.

To elucidate the potential underlying causes of this phenomenon, we selected a subset of females who exhibited co-occurrences of sarcopenia and obesity. This subset was further divided into two subgroups based on the IL-16 cutoff value (150 pg/mL). Only the subgroup with high IL-16 levels showed a significant positive correlation between the BFR and MNA score (r = 0.574, *p* = 0.040). A significant disparity in ASMI scores was also observed between the groups with high and low levels of IL-16 (high IL-16—5.73 kg/m^2^; low IL-16—6.46 kg/m^2^, *p* = 0.010). Taken together with the outcomes from Figure 2, the observed higher fat mass in the female patient group compared with the control group and the absence of a correlation between the BFR and nutritional status in the low-IL-16 subgroup of sarcopenic, obese females, we hypothesize that in women with sarcopenia, an elevated IL-16 level may impede the conversion of nutrients into muscle mass and favor a greater direct conversion of nutrients into fat for storage.

Sarcopenic obesity (SO) was observed in both male and female patient groups, as characterized by concurrent reductions in body weight, FM, and ASMM. However, there was no significant difference in the BFR between the patient and control groups in males. In contrast, the females in the patient group exhibited a substantial decrease in body weight and ASMM, while the FM was higher compared to the control group. Consequently, a significant disparity in the BFR emerged between the two female groups, with the patient group showing significantly higher values than the control group. The increase in BFR is typically the result of multiple factors, including reduced muscle mass, decreased metabolic rate, and excessive energy intake. In our study, the primary reason for the elevated BFR in female individuals with sarcopenia was a notable decline in muscle mass accompanied by an increase in body fat, as fat filled the void left by muscle loss.

IL-6 serum levels were associated with the amount of adipose tissue in multiple body parts [26]. Moreover, increased levels of IL-16 were linked to an ongoing inflammatory response in obese individuals [27]. Thus, elevated IL-16 may also lead to the development of disease conditions that are related to obesity. Hence, IL-16 is a proinflammatory chemokine that induces the migration of immune cells to the site of inflammation. Inflammation is assumed to be the reason for initiating and exacerbating muscle breakdown, and multiple cytokines have been shown to be associated with muscle mass loss. Previous studies have demonstrated that in gastric cancer patients, sarcopenia is associated with IL-16, with higher levels of IL-16 expression detected in gastric cancer patients with sarcopenia [9]. IL-16 may be one of the interleukins that trigger SO in older females. High levels of IL-16 in the body indirectly increase the BFR of female patients by increasing muscle mass loss, thus triggering SO. Female aging is associated with changes in hormonal status, ovarian dysfunction, and the dysregulation of immune functions in various organs, including muscles. Moreover, IL-16 expression rapidly increases during the ovarian aging process in the late menopausal period [15]. Thus, it is conceivable that IL-16 may lead to sarcopenia by changing female hormone levels. In fact, changes in the ASMI with IL-16 level were not significant in males, and there were no differences in the BFR between the patient and control groups. Other factors, such as nutritional status, may play a crucial role in influencing the risk of sarcopenia in males.

## 5. Conclusions

Based on the disparities observed in IL-16 levels and BFRs between the male and female sarcopenic patients in the patient and control groups, as well as the subsequent discussion on sarcopenic obesity in females, our study suggests a sex-specific variation in the pathogenesis of sarcopenia. While nutritional deficiency may directly contribute to sarcopenia in males, the impact of IL-16 appears to be more relevant in female muscle generation. Traditionally, improving nutrition has been considered an effective strategy for reducing the occurrence and progression of sarcopenia. However, our study indicates that this approach may be more effective in males than in females. These findings may have important implications for the enhancement and treatment of sarcopenia in older adults. Nevertheless, our study has certain limitations, and further population-based and laboratory research is necessary to validate these results. Overall, our study presents a novel hypothesis regarding the sex-specific pathogenesis of sarcopenia in older adults and proposes the potential use of IL-16 as a specific marker for the development of sarcopenic obesity in older women.

## Figures and Tables

**Table 1:** Study group characteristics based on sex, analyzed using descriptive statistics.

	Male (*n* = 35)	Female (*n* = 45)	*p*
Age (years)	80.34 ± 7.05	82.31 ± 5.81	0.232
ASMI (kg/m^2^)	6.93 ± 1.07	6.15 ± 0.97	0.001
Weight (kg)	76.27 ± 16.45	67.14 ± 13.55	0.008
Grip Strength (kg)	20.64 ± 10.04	13.84 ± 5.61	0.001
BFR (%)	30.24 ± 8.00	35.93 ± 7.36	0.001
MNA (score)	20.69 ± 5.74	21.36 ± 4.36	0.782
IL-16 (pg/mL)	140.19 ± 83.40	145.91 ± 76.21	0.731

**Table 2:** Characteristics of the patient and control groups, determined using descriptive statistics.

		Control Group Male ≥ 7.3 kg/m^2^ Female ≥ 6.8 kg/m^2^ (*n* = 24)	Patient Group Male < 7.3 kg/m^2^ Female < 6.8 kg/m^2^ (*n* = 56)	*p*
Sex (F/M)		10/14	35/21	0.087
Age (years)	Male	77.79 ± 6.67	82.05 ± 6.92	0.098
	Female	81.80 ± 3.39	82.46 ± 6.37	0.520
Weight (kg)	Male	86.86 ± 12.12	69.21 ± 15.29	0.001
	Female	74.75 ± 12.29	64.97 ± 13.26	0.043
ASMM (kg)	Male	23.54 ± 3.45	17.97 ± 2.82	<0.001
	Female	20.26 ± 1.99	15.54 ± 2.23	<0.001
FM (kg)	Male	26.52 ± 9.21	21.88 ± 10.43	0.051
	Female	23.14 ± 8.76	25.02 ± 8.34	0.682
BFR (%)	Male	29.98 ± 8.87	30.40 ± 7.59	0.840
	Female	29.91 ± 8.10	37.64 ± 6.24	0.002
MNA (score)	Male	23.11 ± 5.28	19.07 ± 5.56	0.039
	Female	25.30 ± 3.74	20.23 ± 3.88	0.001
IL-16 (pg/mL)	Male	181.99 ± 92.25	112.33 ± 65.24	0.013
	Female	98.77 ± 62.20	159.38 ± 75.18	0.025

**Table 3:** Factors influencing the sarcopenia patient group, detected in a multivariate analysis using Fisher’s exact test in males and females.

			OR	95% CI	*p*
MNA (score)	Male	Low (scores < 17)	9.75	1.07–88.87	0.028
		High (scores ≥ 17)			
	Female	Low (scores < 17)	4.63	0.24–89.42	0.312
		High (scores ≥ 17)			
BFR (%)	Male	Obesity (≥25.0%)	1.28	0.28–5.93	0.752
		Normal (<25.0%)			
	Female	Obesity (≥35.0%)	5.83	1.25–27.17	0.027
		Normal (<35.0%)			
IL-16 (pg/mL)	Male	High (≥150 pg/mL)	0.18	0.04–0.81	0.031
		Low (<150 pg/mL)			
	Female	High (≥150 pg/mL)	9.53	1.09–83.44	0.029
		Low (<150 pg/mL)			

## Data Availability

The data presented in this study are openly available at https://www.ukaachen.de/en/kliniken-institute/klinik-fuer-altersmedizin-med-klinik-vi/forschung/biogerontology/sarcopenia/ (accessed on 8 August 2023).

## Supplementary Materials

The following supporting information can be downloaded at: https://www.mdpi.com/article/10.3390/nu15163529/s1, Table S1: Detected levels of cytokines/chemokines (pg) in the serum samples from all study participants (mL).
