# Supplementary material for: Sex-Specific Associations between Serum IL-16 Levels and Sarcopenia in Older Adults
Source: Nutrients. 2023 Aug 10;15(16):3529. doi: 10.3390/nu15163529 (PMC10459512; doi:10.3390/nu15163529)
Supplement: Supplementary file 1 [file nutrients-15-03529-s001.zip › nutrients-2528460-supplementary.pdf]

Supplementary Table.S1 Detected levels of cytokines/chemokines in the serum samples from all study participants (pg/ml). Data of study groups based on different ASMI cut-off values analyzed using descriptive statistics. GDF2 was not delectable in all samples (ND).

		**Control group** (*n* = 24)	**Case group** (*n* = 56)	*p*
TNFSF13	Male	3201.96±22456.69	2908.84±1504.67	0.711
	Female	3272.84±1761.21	2422.09±1233.46	0.161
TNFSF13B	Male	2.12±1.75	2.65±2.07	0.294
	Female	2.81±2.32	2.26±2.32	0.243
BDNF	Male	79.79±73.73	60.04±59.28	0.385
	Female	86.21±61.19	70.12±58.00	0.263
GDF2	Male	ND	ND	ND
	Female			
EGF	Male	44.17±33.83	37.53±23.48	0.662
	Female	36.05±35.57	51.68±35.72	0.128
CCL24	Male	272.80±202.16	217.14±113.09	0.788
	Female	229.08±114.00	252.37±111.85	0.604
FABP-3	Male	2220.05±1426.11	2666.85±2271.41	0.822
	Female	3359.66±3101.11	2616.19±1976.12	0.397
CNPY2	Male	12.01±9.60	16.65±10.42	0.398
	Female	15.45±11.47	11.67±8.00	0.542
ILCF	Male	39.33±21.69	43.42±22.24	0.471
	Female	53.13±26.68	40.44±17.26	0.202
HGF	Male	200.86±133.85	206.96±155.68	0.854
	Female	199.51±135.92	232.55±194.75	0.801
IL-2R	Male	3795.60±3324.22	2790.89±2808.14	0.202
	Female	2486.01±1695.64	3530.12±2290.41	0.172

IL-13	Male	3.09±2.59	4.37±2.35	0.192
	Female	5.89±4.87	4.05±3.46	0.444
IL-16	Male	181.99±92.25	112.33±65.24	0.013
	Female	98.77±62.20	159.38±75.18	0.025
IL-20	Male	9.43±16.19	14.29±21.79	0.552
	Female	6.63±7.94	11.26±21.38	0.954
LIF	Male	28.05±18.99	26.55±12.90	0.840
	Female	40.40±16.27	25.32±13.78	0.007
ALOX15	Male	53.74±44.21	57.38±42.80	0.632
	Female	55.05±54.19	54.10±40.61	0.623
CCL8	Male	7.19±2.85	6.94±3.80	0.614
	Female	6.26±3.46	6.39±3.02	0.716
CCL7	Male	35.83±22.28	39.41±19.31	0.512
	Female	47.22±21.11	35.77±19.66	0.101
MDC	Male	93.80±40.39	74.20±41.58	0.195
	Female	99.03±47.92	88.05±45.17	0.556
MIF	Male	36.05±19.10	40.46±21.18	0.381
	Female	36.83±12.43	41.79±18.79	0.512
CXCL9	Male	27.12±19.24	53.64±56.82	0.312
	Female	36.50±25.46	39.10±28.55	0.955
MMP-1	Male	1017.48±848.30	736.61±672.35	0.312
	Female	950.41±743.85	908.65±839.42	0.818
MMP-13	Male	23.01±16.88	23.70±16.48	0.897
	Female	24.98±16.87	18.07±13.97	0.287
NGF β	Male	2.14±3.14	1.95±2.10	0.428
	Female	1.95±1.36	1.47±1.65	0.101

PDGF-BB	Male	175.57±54.02	177.76±105.69	0.699
	Female	134.39±83.63	121.02±114.65	0.424
PlGF-1	Male	14.16±17.35	19.02±20.64	0.321
	Female	13.73±10.63	14.33±15.48	0.782
SCF	Male	11.56±5.53	9.97±7.04	0.439
	Female	12.96±9.16	12.27±7.79	0.859
TNF-RII	Male	168.36±80.62	152.25±61.73	0.637
	Female	170.99±49.35	162.07±65.00	0.634
VEGFA	Male	873.15±800.37	693.66±461.40	0.946
	Female	767.57±526.11	780.49±650.49	0.758
VEGFD	Male	4.91±2.80	5.37±3.62	0.730
	Female	8.02±5.50	6.94±6.52	0.249
ICAM-1	Male	56011.18±36336.52	50566.26±31886.30	0.713
	Female	50637.96±24118.06	70510.10±48518.62	0.263
MMP-3	Male	659.26±337.26	732.63±307.73	0.329
	Female	647.29±348.23	739.16±369.46	0.484
MMP-9	Male	3570.88±1909.73	3156.19±2020.59	0.567
	Female	2382.85±1659.76	3462.13±2020.42	0.133
VCAM-1	Male	23271.60±14509.26	18730.00±94454.78	0.423
	Female	20852.71±18369.49	18016.40±9909.41	0.956
